# Overcoming Therapeutic Inertia in Type 2 Diabetes Care—Timing, Context, and Appropriateness of Treatment Intensification

**DOI:** 10.1001/jamanetworkopen.2021.30926

**Published:** 2021-10-01

**Authors:** Rozalina G. McCoy, Patrick J. O’Connor

**Affiliations:** Division of Community Internal Medicine, Department of Medicine, Mayo Clinic, Rochester, Minnesota; Division of Health Care Delivery Research, Mayo Clinic Robert D. and Patricia E. Kern Center for the Science of Health Care Delivery, Rochester, Minnesota; HealthPartners Institute, Minneapolis, Minnesota

Timely, evidence-based, and safe control of hyperglycemia is foundational to the management of type 2 diabetes and is essential for preventing acute and chronic complications of this common and serious disease. There are 2 main reasons for suboptimal glycemic control: the patient’s inability, for whatever reason, to adhere to prescribed medication(s) and the clinician’s failure to initiate or intensify glucose-lowering therapy when it is clinically appropriate to do so (ie, therapeutic inertia). Therapeutic inertia is common, affecting as many as 50% of patients with type 2 diabetes,^1^ and is driven by a wide range of barriers at the clinician, patient, and health system levels.^2^ Addressing therapeutic inertia is a priority viewed as central to reducing the burden of diabetes and its complications.^2^

Nearly 30% of adults aged 65 years and older are living with diabetes.^3^ Glycemic control among older adults is generally better than among younger patients; recent population-based estimates revealed that 24% of older adults without diabetes complications had glycated hemoglobin (HbA_1c_) levels of 7.5% or greater (ie, the commonly accepted upper threshold for healthier older adults [to convert to proportion of total hemoglobin, multiply by 0.01]), while 20% of older adults with diabetes complications had HbA_1c_ levels of 8.0% or greater^3^ (ie, the recommended upper threshold for more clinically complex patients), suggesting that there is an opportunity to improve glycemic management and reduce the burden of diabetes complications. Hospitalization may be viewed as an opportunity to address therapeutic inertia and improve glycemic control. However, as demonstrated by Anderson et al^4^ elsewhere in *JAMA Network Open*, intensification of glucose-lowering therapy upon hospital discharge does not appear to improve glycemic control but exposes patients to risk of preventable harm due to severe hypoglycemia in the immediate postdischarge period.

As observed by Anderson et al^4^ in a large retrospective cohort study of patients with non–insulin-treated type 2 diabetes aged 65 years or older who were admitted to US Veterans Health Administration hospitals for a wide range of routine medical problems unrelated to diabetes, having glucose-lowering medications intensified at or before hospital discharge was associated with a 2-fold increase in the rate of severe hypoglycemic events requiring emergency department or hospital care during the initial 30-day period after hospital discharge. There was, however, also a significant reduction in 30-day all-cause mortality (hazard ratio, 0.55; 95% CI, 0.33–0.92), which the authors attribute to unmeasured confounding and lower perceived risk of death among patients who were deemed eligible for treatment intensification. This mortality benefit was limited to patients with preadmission HbA_1c_ levels greater than 7.5% (ie, uncontrolled diabetes) and was no longer apparent after 1 year of follow-up. Indeed, after 1 year, there was no association between glucose-lowering medication intensification and severe hypoglycemic or hyperglycemic events, HbA_1c_ level, all-cause hospitalizations, or mortality.

Depending on one’s perspective, several conclusions can be drawn from this study. On the one hand, treatment intensification at transitions of care appears to be ill advised. Intensification of glucose-lowering medications during hospitalization for causes unrelated to diabetes—the condition whose treatment is being intensified—does not improve glucose control as measured by HbA_1c_ level. Concurrently, it transiently doubles the risk of severe hypoglycemia as patients begin to take their newly prescribed medications, nearly all of which in this study were associated with heightened hypoglycemia risk (ie, insulin and sulfonylurea). On the other hand, treatment intensification may be beneficial, as patients receiving intensified treatment were nearly half as likely to die in that same period. While the finding of decreased short-term mortality in the intensified group may be due to confounding, as the authors suggest, other plausible explanations exist. For example, patients receiving treatment intensification may have closer follow-up in the outpatient setting or derive benefit from improved glycemic control with respect to recovery from illness, infection risk, and wound healing. HbA_1c_ is an imperfect measure of glycemic control given that it overlooks the impacts of real-time glucose levels, glycemic variability, and presence of severe and/or symptomatic hypoglycemia and hyperglycemia, factors that can all lead to morbidity in patients with diabetes.

Another key finding of the study by Anderson and colleagues^4^ is the poor rate of persistence to newly prescribed medications. Overall, 34.5% of newly prescribed medications were filled only once—at hospital discharge—and never filled again. By 1 year, only 52.0% of patients who had started oral medications and 61.4% of patients who had started insulin were still taking these drugs. This can reflect the fact that prescribed medications were not truly necessary but rather were prescribed for self-limited hyperglycemia or based on assumptions of poor glycemic control stemming from inpatient hyperglycemia and acute illness. These findings further underscore the need for better integration of hospital and ambulatory care, medication reconciliation and review during posthospital follow-up appointments, and comprehensive transitions-of-care programs.

Several factors limit the interpretation of these data. The observational study design precludes causal inference, and a further limitation is the inability to assess the impact of insulin dose adjustments that are likely more prevalent than adding a new type of insulin. Some of the treatment intensifications may not have been clinically appropriate; an earlier study in the same population found that 49% of patients whose treatment regimens were intensified either had limited life expectancy or were already at their individualized HbA_1c_ goal.^5^ The rate of serious hypoglycemic events is underestimated because it excludes functionally and clinically significant hypoglycemia that did not lead to emergency department or inpatient treatment. Medication intensification is defined as the introduction of a new class of medication or an increase in the dose of an existing noninsulin medication, which would misclassify substitution of 1 glucose-lowering medication for another as an intensification. Finally, few study subjects were receiving sodium-glucose transport protein 2 (SGLT2) inhibitor or glucagon-like peptide-1 (GLP-1) receptor agonist medications either before or after their index hospitalizations, which occurred from 2011 to 2016; nearly all patients were started on insulin or sulfonylureas.

However, the world has changed a lot since 2016. Emerging evidence and current diabetes clinical guidelines advise that many patients with type 2 diabetes and established cardiovascular or kidney disease may benefit from adjusting medication regimens to include SGLT2 inhibitor and/or GLP-1 receptor agonist medications, which did not occur in this study. Initiation of these medication classes in patients such as those in this study, of whom more than half had coronary artery disease and kidney disease and more than one-third had heart failure, may well have resulted in lower rates of hypoglycemia, hospitalizations, and deaths.

In this context, a new set of research questions emerges. First, which patients are appropriate candidates for safe and effective treatment intensification, and with which medications? SGLT2 inhibitors and GLP-1 receptor agonists should be considered for patients with high risk of cardiovascular disease, but what about patients with moderate levels of risk? Second, what is the optimal timing and clinical context for treatment intensification when such action is clinically appropriate? Third, how can we best inform patients of the benefits and risks of alternative care options to effectively engage in shared decision-making that may improve medication adherence? Fourth, better coordination of care between inpatient and outpatient settings deserves our attention. Addressing therapeutic inertia and making care transitions safer require ongoing innovation and testing a wide variety of potential improvement strategies, ranging from improvement in health informatics systems to the use of social workers or nurses to facilitate communication and coordination of care around the time of hospital discharge. These efforts are commendable, but ultimately their success depends on a proactive, resilient primary care system that is well integrated with hospital care.^6^
